# Effects of Exogenous Putrescine on Delaying Senescence of Cut Foliage of *Nephrolepis cordifolia*

**DOI:** 10.3389/fpls.2020.566824

**Published:** 2020-09-10

**Authors:** Ying Qu, Lu Jiang, Tana Wuyun, Shouyuan Mu, Fuchun Xie, Yajun Chen, Lu Zhang

**Affiliations:** ^1^ College of Horticulture and Landscape Architecture, Northeast Agricultural University, Harbin, China; ^2^ Chair of Crop Science and Plant Biology, Estonian University of Life Sciences, Tartu, Estonia

**Keywords:** antioxidant enzyme, biochemical, chlorophyll fluorescence imaging, polyamines, sword fern

## Abstract

Senescence is the main limitation for cut foliage display in vase. Naturally occurring polyamines such as putrescine (Put) have been considered effective anti-senescence agents. However, effect of Put on cut foliage in vase in a realistic indoor environment has not yet been revealed. In the present study, effects of Put spraying on the postharvest performance of cut foliage of *Nephrolepis cordifolia* L. were investigated. Cut fronds sprayed with deionized water (Put0) showed visible injuries after 10 days in vase. Meanwhile, chlorophyll (Chl), soluble protein (Sp), and proline (Pro) content were decreased by 60.15, 57.93, and 73.09% respectively, photochemical activity reflected by Chl fluorescence parameters was inhibited, whereas electrolyte leakage (EL), contents of soluble sugar (Ss), malondialdehyde (MDA), and hydrogen peroxide (H_2_O_2_) were increased (+194.29, +44.83, +34.06, and +178.01%, respectively). Put spraying extended the vase life of the cut foliage and the 2.0 mM Put had a longer vase life (21 days) than 0.2 mM (15 days). Leaf spraying of 2.0 mM Put for 10 days significantly ameliorated the losses of Chl, Sp, and Pro content (−10.72, −26.29, and −42.64%, respectively), followed by 0.2 mM Put (−27.36, −36.24, and −60.55%, respectively). Put spraying also improved photochemical capability and prevented membrane impairment as well as visible injury in comparison with Put0. In addition, 2.0 mM Put had a better mitigating ability than that of 0.2 mM. Leaf spraying of 2.0 mM Put greatly reduced the decline of the effective quantum yield of photochemical energy conversion in PSII (ΦPSII), the maximal quantum yield of PSII photochemistry measured in the dark-adapted state (Fv/Fm) and electron transport rate (ETR) (−7.89, −12.91, and −10.06%, respectively), and also inhibited the increases of EL, MDA, Ss, and H_2_O_2_ (+31.87, +6.43, +16.22, and +49.40%, respectively). Overall, Put played important roles in deterring the degradation of Chl, Ss, and Pro, detoxifying the H_2_O_2_, weakening the sugar signaling, mitigating the decline of photochemical activity, and eventually postponing the leaf senescence. The present study gives new insights into effects of Put on leaf senescence and provides a strategy for preserving post-harvest cut foliage.

## Introduction

In recent decades, with continuous improvement of standard of living and increasing desire for a better indoor environment, indoor greening and decoration has emerged, with a strong demand for cut flowers and foliage. The short vase life and the low ability to cope with unsuitable environment during floral arrangement and display are the most important constraints for the commercialization of cut flowers and foliage (Zamani et al., 2011; Zhao et al., 2017).

For cut flowers and foliage, senescence is the main reason for short vase life (Shabanian et al., 2018). Although cut foliage plays important roles in floriculture industry, studies on its postharvest senescence are very limited compared with cut flowers (Malakar et al., 2017; Che-Husin et al., 2018; Lin et al., 2019). Senescence caused by the detachment of leaves from plants is associated with various biochemical and physiological changes. Leaf yellowing is often the first visible symptom of senescence, mainly due to the loss of chlorophyll (Chl) (Skutnik et al., 2004). It has been reported that after leaves were detached from rabbit’s foot fern (*Phlebodium aureum*), Chl *a* and Chl *b* contents decreased (Teerarak and Laosinwattana, 2019). Chl breakdown often occurs due to the remobilization of nitrogen from Chl-binding proteins to other processes during leaf senescence (Hörtensteiner, 2006). Besides Chl, protein is also considered as a marker of senescence since it degrades during leaf senescence (Breeze et al., 2011). Furthermore, many studies have found that reactive oxygen species (ROS) level increases during leaf senescence (Merzlyak and Hendry, 1994; Breusegem and Dat, 2006). Plants orchestrate a myriad of antioxidants such as ascorbic acid (AsA) as a strategy to cope with the detrimental ROS. For instance, it has been observed that increases in peroxidase (POD) and superoxide dismutase (SOD) activity occur in senescing leaves (Kim et al., 2004). Moreover, senescence induces an increase in the content of polyamines such as putrescine (Put), spermidine (Spd), and spermine (Spm) (Serrano et al., 2001; Sobieszczuk-Nowicka et al., 2016). Study on the maximum quantum yield of photosystem II (PSII) photochemistry measured in the dark-adapted state (Fv/Fm) shows that it decreases in *Sorghum bicolor* during leaf senescence (Chen et al., 2015).

Polyamines (PAs) are biodegradable organic compounds and have no adverse environmental effects (Sharma et al., 2017). Naturally occurring PAs such as Put have been reported as effective anti-senescence agents and lower Put content was found in aged cell (Paschalidis and Roubelakis-Angelakis, 2005; Kusano et al., 2008). The addition of PAs (Put, Spd, and Spm) increases the longevity of isolated flowers of *Nicotiana plumbaginifolia* (Nisar et al., 2015). Conditioning of cut stems of rose in Put solution results in a higher Chl *a* and Chl *b* content (Rubinowska et al., 2012). Exogenously supplied Put reduces hydrogen peroxide (H_2_O_2_) accumulation, electrolyte leakage (EL) and malondialdehyde (MDA) content, while increasing the activities of catalase (CAT) and ascorbate peroxidase (APX), thereby prolonging the vase life of cut lisianthus flowers (Ataii et al., 2015). Leaf senescence is different from petal senescence. For example, cut foliage can still sustain photosynthesis and do not need energy to maintain its survival at the late stage of senescence process (Zhang and Becker, 2015). However, information about the effects of PAs on cut foliage is very limited. In the limited studies, scientists focused on the roles of PAs in Chl and protein loss and antioxidant system with or without light (Altman, 1982; Cho and Hong, 1988). Generally, these studies have uncoupled the dark and light responses to investigate the senescence mechanisms. However, to reveal how Put affects postharvest quality of cut foliage, the holistic effects on leaf senescence should be considered in a realistic light-dark cycle. To our best knowledge, relevant reports are very few (Sood and Nagar, 2003). Furthermore, the state of art technology supplies novel insights to reveal the mechanism of leaf senescence. For example, Chl fluorescence imaging provides a non-destructive method to detect the photochemical damage in a whole leaf scale and very suitable for studying the anti-aging effect of Put. However, this method has not yet been applied in this field.

Sword fern (*N. cordifolia* L.) is commonly used in the garden and its cut green foliage has a high decorative value in floral arrangements due to it providing a vertical accent to floral designs (Safeena et al., 2019). In addition, it is available throughout the year. Therefore, in the present study, we selected sword fern as plant material and aimed to investigate the potential mitigating effects of Put on the senescence of cut foliage under realistic indoor environment and to reveal the underlying physiological and biochemical mechanisms. These results could provide a theoretical basis for prolonging the vase life of cut foliage using exogenous Put.

## Materials and Methods

### Plant Material

Sword fern (*N. cordifolia* L.) was selected as plant material. A total of 50 pots of plants were bought from Harbin Flower Market, CN. The diameter of the pots was 16 cm and the average height of the plants was ca. 30 cm. Before treatments, the plants were adapted for one week in a greenhouse where the average temperature was ca. 25 °C and relative humidity ca. 65%. During this period, the light period was 16/8 h (day/night), and the average light intensity was ca. 800 µmol (photon) m^-2^ s^-1^. The plants were irrigated to the soil water capacity every day.

### Treatment and Vase Life Observation

A total of 72 healthy fronds with similar size (ca. 20 cm long, in which both the lamina and the stipe were 10 cm) were cut from the plants using a sharp knife, then each frond was immediately placed upright into one individual conical flask containing 100 ml of deionized water. The conical flasks were covered with transparent plastic films to decrease water evaporation and placed on a table in the laboratory. Fronds together with conical flasks were randomly and equally divided into four groups (18 cut fronds for each group). For the control group, the cut fronds were observed and sampled immediately, then the samples were stored in -80 °C for the following measurements. The other three groups were sprayed with 0.2 mM Put (Put0.2), 2.0 mM Put (Put2.0), or deionized water (Put0), respectively. The Put concentrations were selected based on references (Yiu et al., 2009; Rubinowska et al., 2012) and a preliminary assay. During the preliminary assay, we used 0.2, 2.0, 4.0 mM Put to test whether they can extend the vase life of cut fronds and found that the frond sprayed with 4.0 mM Put had a shorter vase life than that with Put0 (data not shown). Spraying treatments (2 ml for each frond) were conducted every day in the morning (09:00 am) for consecutive 10 days till 50% of the fronds in Put0 group showed leaflet drop. Then 12 cut fronds of each group were observed and sampled immediately. The samples were also stored in -80 °C for the following measurements. The remaining six cut fronds in each group continue to be sprayed as before to observe the vase life. When 50% of the fronds in each group showed leaflet drop, the vase life was recorded. During the treatment period, the range of relative humidity was ca. 40–55%, the temperature ca. 28/24 °C (day/night), the light period 16/8 h (day/night), and the average light intensity ca. 100 µmol (photon) m^-2^ s^-1^.

### Leaf Appearance

The cut foliage was photographed before and after 10 days of treatment in different groups. Six intact fronds in each treatment group were selected. The percentage of injured area on each cut frond was observed and recorded by three surveyors using a 5 and 1% step scale independently (Paoletti et al., 2009).

### Chl Content

Fresh leaflet (0.3 g, n = 6) was sampled and ground with quartz sand and ethanol (95%, v/v), then the extract was filtered into a 25 ml brown volumetric flask, in which the volume was kept at 25 ml using ethanol (95%, v/v). Absorbance of the extracting solution was measured at 470, 649, 665 nm using an Ultraviolet-Visible spectrophotometer (*T6 New Century*, CN). Contents of Chl *a*, Chl *b*, and total Chl, were calculated according to Scrob et al. (2019).

### Membrane Integrity

EL was determined according to the method of Lutts et al. (1996). Fresh leaf sample (0.1 g, n = 6) was put into test tubes containing 10 ml of deionized water at 25 °C. After 24 h, EL was determined using a conductivity meter as EL_1_. Then samples were maintained in boiling water bath (100 °C) for 20 min. After cooling to 25 °C, a last EL reading (EL_2_) was obtained. The EL was defined as: EL_1_/EL_2_×100%.

Fresh leaflet sample (0.15 g, n = 6) was ground with trichloroacetic acid (10%). Then the homogenate was centrifuged at 4,000×g for 10 min. The supernatant (1 ml) was mixed with 1 ml of thiobarbituric acid (0.6%) and then maintained in boiling water bath for 15 min. After cooling, the mixture was centrifuged at 4,000×g for 10 min. The absorbance of supernatant was detected at 450, 532, 600 nm using an Ultraviolet-Visible spectrophotometer (*T6 New Century*, CN). The MDA content was calculated according to Li et al. (2018).

### Reactive Oxygen Species (ROS)

Fresh leaflet sample (0.5 g, n = 6) was put in an ice-cold mortar and ground with 50 mM phosphate extraction buffer (PBS, 3 ml, pH 7.8). Thereafter the homogenate was centrifuged at 12,000×g for 15 min at 4 °C and the supernatant was used to measure the content of superoxide anion (O_2_^• −^). The content of O_2_^• −^ was determined according to the method of Tian et al. (2003). The concentration of H_2_O_2_ was determined by the method of Patterson et al. (1984). To determine the H_2_O_2_ content, the leaflet sample (1.0 g, n = 6) was put in a mortar and ground in cold acetone (5 ml). The homogenate was centrifuged at 5,000×g for 5 min at 4 °C and the supernatant was used to determine the content of H_2_O_2_.

### Antioxidant System

Preparation of SOD and CAT sample solution is the same as O_2_^• −^. SOD activity (U g^-1^ fresh weight, FW) was quantified according to the method of Syeed et al. (2011). One unit of SOD activity (U) was defined as the amount of enzyme that inhibited the photoreduction of 50% nitro blue tetrazolium (NBT). Activity of CAT was quantified based on the method described by Díaz-Vivancos et al. (2008). One unit of CAT activity represented the amount of enzyme catalyzing the decomposition of 1 µmol (H_2_O_2_) min^-1^ g^-1^ FW. Ascorbic acid (AsA) content was measured according to Law et al. (1983). The method is based on the reduction of Fe^3+^ to Fe^2+^ by AsA in acidic solution. Then Fe^2+^ forms complexes with bipyridyl, which appears pink at an absorption of 525 nm.

### Cellular Macromolecules

Soluble sugar (Ss), soluble protein (Sp), and proline (Pro) levels were estimated. Anthrone-sulfuric acid method (Leng et al., 2016) was employed to measure Ss. Finally, the absorbance of solution was detected at 620 nm. The Sp content was measured based on the binding of Coomassie Brilliant Blue G-250 to protein according to the Bradford method (Bradford, 1976). Fresh leaf (0.5 g, n = 6) was homogenized using 3% sulfosalicylic acid solution (5 ml) and then maintained in boiling water bath (100 °C) for 10 min (stirred often). After cooling, Pro extract was obtained by filtering the mixture. According to the method (Teklić et al., 2010), the supernatant was used to determine the Pro content.

### Chl a Fluorescence

In the early morning of sampling date, Chl *a* fluorescence of cut frond was measured using an IMAGING-PAM Chlorophyll Fluorometer (*Heinz* *Walz*, Effeltrich, Germany). Six intact fronds per treatment were used. The selected cut fronds were adapted in the dark for 20 min. Thereafter fluorescence induction curves were automatically recorded using the slow kinetic program in the saturation pulse analysis mode. The values of the minimal fluorescence yield in the dark-adapted state (Fo) and the maximal fluorescence yield of the dark-adapted state (Fm) were obtained with a modulated light [650 nm, 0.5 µmol (photon) m^-2^ s^-1^] and a 0.8 s saturating pulse [650 nm, 3,700 µmol (photon) m^-2^ s^-1^], respectively. After 40s, an actinic light irradiation [650 nm, 196 µmol (photon) m^-2^ s^-1^] was turned on. Then, a saturating pulse was imposed every 20 s until 5 min to determine the maximal fluorescence in the irradiation-adapted state (Fm’). Fv/Fm was calculated as: Fv/Fm=(Fm−Fo)/Fm (Kitajima and Butler, 1975). Stern-Volmer non-photochemical quenching coefficient (NPQ), coefficient of photochemical quenching of variable fluorescence based on the puddle model of PSII (qP), effective quantum yield of photochemical energy conversion in PSII (ΦPSII), quantum yield of regulated energy dissipation in PSII (ΦNPQ), quantum yield of non-regulated energy dissipation in PSII (ΦNO), and electron transport rate (ETR) were calculated by the ImagingPamGigE software (*Heinz Walz*, Effeltrich, Germany). Values of four circular areas (from the top to the bottom positions) on each cut frond were recorded, then averaged as a replicate for statistical analysis.

Images of the fluorescence parameters were stored in the ImagingPamGigE software and displayed by means of a false color code ranging from black (0.0) to purple (ending at 1.0) *via* red, yellow, green, and blue.

### Statistical Analysis

All data were analyzed using SPSS (v.12, SPSS, Chicago, IL, USA). One-way analysis of variance (ANOVA) was used to identify the effect of Put solution. Post-hoc Duncan’s test was used to compare means of each parameter among different treatments. The relative effect of Put spraying on each parameter was calculated as [(Value_treatment_–Value_control_)/Value_control_].

## Results

### Vase Life of Cut Foliage

According to the experimental observation, when sprayed with deionized water for 10 days, 50% of the cut fronds in the deionized water group showed leaflet drop, whereas the vase life was extended by 5 days in Put0.2 group and by 11 days in Put2.0 group.

### Leaf Appearance and Visible Symptoms

**Figure 1:**
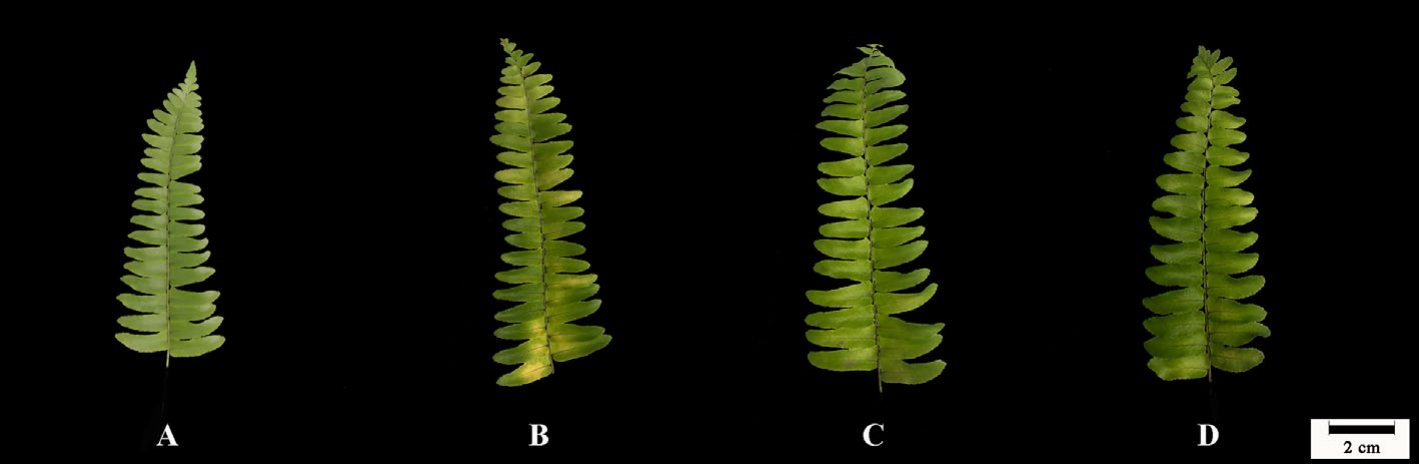
Leaf appearance of cut foliage of *N. cordifolia* before (Control, **A**) and after 10 days spraying with deionized water (Put0, **B**), 0.2 mM Put (Put0.2, **C**), and 2.0 mM Put (Put2.0, **D**).

The foliage before treatment (Control) exhibited no visible injury (**Figure 1**). After 10 days of treatment, six cut fronds were starting to shed the leaflets and the other six had chlorotic blades under Put0 (15.92% of the total fronds area). Compared with Put0 treatment, the average percentages of visible yellowing area over the leaf surface under Put0.2 and Put2.0 were significantly lower, 2.40 and 1.18%, respectively (**Table 1**).

**Table 1:** Visible injury on the cut foliage of *Nephrolepis cordifolia* before (Control) and after 10 days spraying with deionized water (Put0), 0.2 mM Put (Put0.2), and 2.0 mM Put (Put2.0).

Treatment	Visible injury (%)
Control	0
Put0	15.92 ± 0.61^a^
Put0.2	2.40 ± 0.22^b^
Put2.0	1.18 ± 0.14^c^

Data were shown as means ± S.E. (n = 6). Values with different letters indicate significant differences between treatments (Duncan’s test, p ≤ 0.05).

### Chl Content

**Figure 2:**
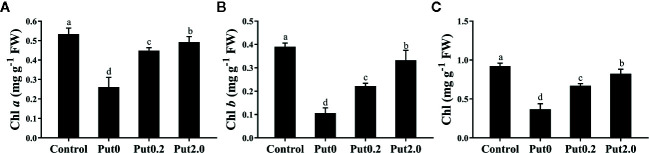
Mean (+ S.E., n = 6) contents of chlorophyll (Chl) *a* **(A)**, Chl *b* **(B)**, and total Chl **(C)**, of cut foliage of *N. cordifolia* before (Control) and after 10 days spraying with deionized water (Put0), 0.2 mM Put (Put0.2), and 2.0 mM Put (Put2.0). FW is the fresh weight of the sample. Different letters show significant differences among bars (Duncan’s test, p ≤ 0.05).

After 10 days of treatment, Chl *a* (−50.98%), Chl *b* (−72.69%), and total Chl (−60.15%) content of cut leaves under Put0 decreased significantly compared with control (**Figure 2**). Put2.0 spraying greatly alleviated the losses of Chl *a* (−7.68%), Chl *b* (−14.86%), and total Chl (−10.72%). Cut fronds sprayed with Put0.2 also had higher Chl *a* (−15.89%), Chl *b* (−43.05%), and total Chl content (−27.36%) than those sprayed with deionized water (Put0), further supporting its anti-senescence effect.

### Membrane Integrity

**Figure 3:**
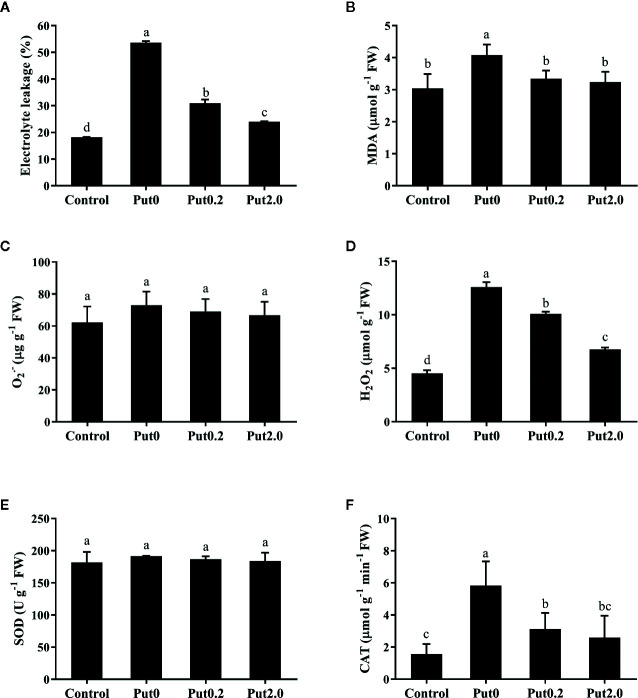
Electrolyte leakage **(A)**, malondialdehyde content (MDA, **B**), superoxide anion content (O_2_^• −^, **C**), hydrogen peroxide content (H_2_O_2_, **D**), and activities of superoxide dismutase (SOD, **E**) and catalase (CAT, **F**) of cut foliage of *N. cordifolia* before (Control) and after 10 days spraying with deionized water (Put0), 0.2 mM Put (Put0.2), and 2.0 mM Put (Put2.0). FW is the fresh weight of the sample. Data were shown as means + S.E. (n = 6). Different letters show significant differences among bars (Duncan’s test, p ≤ 0.05).

Compared to control, fronds sprayed with deionized water had significantly higher EL (+194.29%, **Figure 3A**). The relative increase of EL in leaves sprayed with Put0.2 and Put2.0 were lower (+69.90 and +31.87%, respectively), showing a better membrane integrity. Fronds sprayed with deionized water had a higher MDA (+34.06%) in comparison to control (**Figure 3B**), whereas in the leaves sprayed with Put (Put0.2 and Put2.0), MDA levels were not significantly different from control, indicating that Put could protect the plasma membrane from the ROS attack.

### Reactive Oxygen Species (ROS)

There was no significant difference of O_2_^• −^ content between treatments (**Figure 3C**). H_2_O_2_ content significantly increased in the fronds treated with deionized water (+178.01%) compared to control. Put0.2 and Put2.0 inhibited the increase of H_2_O_2_ (+122.89 and +49.40%, respectively) (**Figure 3D**).

### Antioxidant System

**Figure 4:**
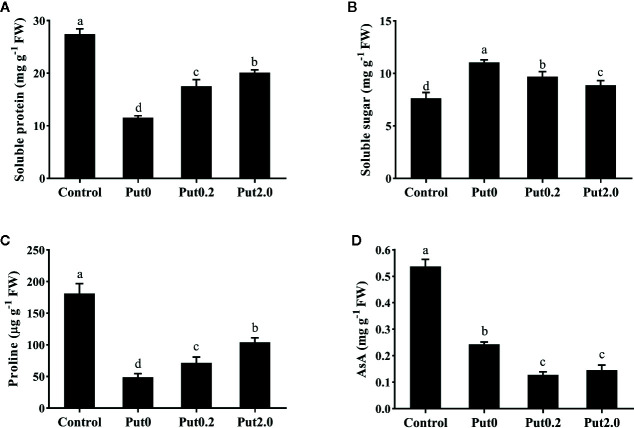
Soluble protein **(A)**, soluble sugar **(B)**, proline **(C)**, and ascorbic acid (AsA, **D**) contents of cut foliage of *N. cordifolia* before (Control) and after 10 days spraying with deionized water (Put0), 0.2 mM Put (Put0.2), and 2.0 mM Put (Put2.0). FW is the fresh weight of the sample. Data were shown as means + S.E. (n = 6). Different letters show significant differences among bars (Duncan’s test, p ≤ 0.05).

There was no significant change in SOD activity between treatments (**Figure 3E**). Compared to control, fronds sprayed with deionized water had significantly higher CAT activity (+273.33%, **Figure 3F**) and Put spraying inhibited the increase. The relative increase of CAT activity in the Put2.0-sprayed (+66.67%) fronds were the lowest, followed by the Put0.2-sprayed fronds (+100%). After 10 days of treatment, AsA contents of cut fronds under Put0, Put0.2, and Put2.0 decreased significantly compared with control (−54.71, −76.24, and −72.87%, respectively) (**Figure 4D**). However, the AsA content was not significantly different between Put0.2 and Put2.0.

### Cellular Macromolecules

After 10 days of treatment, Sp (−57.93%) and Pro (−73.09%) content of cut fronds under Put0 decreased significantly compared with control (**Figure 4**). Put2.0 spraying greatly alleviated the losses of Sp (−26.29%) and Pro (−42.64%). Cut fronds sprayed with Put0.2 also had lower losses of Sp (−36.24%) and Pro content (−60.55%). Cut fronds sprayed with deionized water had higher Ss content (+44.83%, **Figure 4B**) than control and Put spraying inhibited the increase. The relative increase of Ss content in the Put2.0-sprayed fronds was the lowest (+16.22%), followed by the Put0.2 (+26.92%).

### Chl a Fluorescence

**Figure 5:**
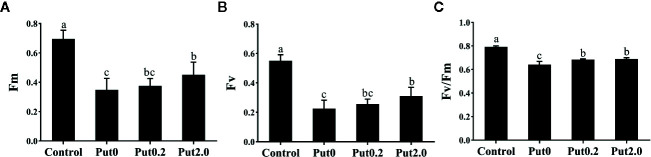
The maximal fluorescence yield of the dark-adapted state (Fm, **A**), variable fluorescence (Fv, **B**), the maximal quantum yield of PSII photochemistry measured in the dark-adapted state (Fv/Fm, **C**) of cut foliage of *N. cordifolia* before (Control) and after 10 days spraying with deionized water (Put0), 0.2 mM Put (Put0.2), and 2.0 mM Put (Put2.0). Data were shown as means + S.E. (n = 6). Different letters show significant differences between bars (Duncan’s test, P ≤ 0.05).

**Figure 6:**
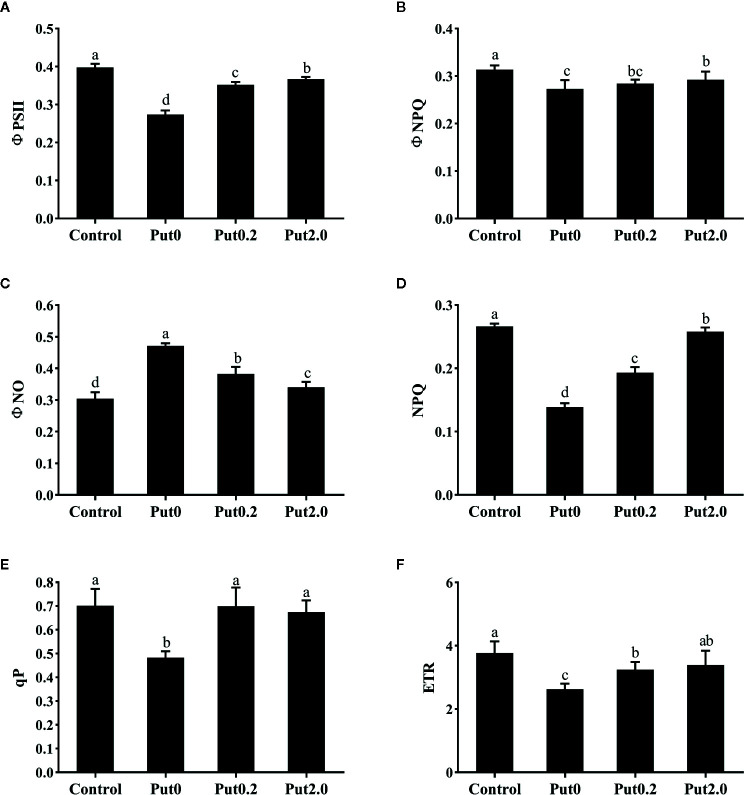
The effective quantum yield of photochemical energy conversion in PSII (ΦPSII, **A**), quantum yield of regulated energy dissipation in PSII (ΦNPQ, **B**), quantum yield of non-regulated energy dissipation in PSII (ΦNO, **C**), non-photochemical quenching coefficient (NPQ, **D**), coefficient of photochemical quenching of variable fluorescence based on the puddle model of PSII (qP, **E**), and electron transport rate (ETR, **F**) of cut foliage of *N. cordifolia* before (Control) and after 10 days spraying with deionized water (Put0), 0.2 mM Put (Put0.2), and 2.0 mM Put (Put2.0). Data were shown as means + S.E. (n = 6). Different letters show significant differences between bars (Duncan’s test, P ≤ 0.05).

Both Fm (−50.02%) and variable fluorescence (Fv, −59.12%) decreased in the fronds under Put0 after 10 days of spraying with deionized water in comparison to control, whereas these values were higher in the fronds sprayed with Put0.2 (−46.05 and −53.34%, respectively) and Put2.0 (−35.19 and −43.55%, respectively) relative to Put0 (**Figure 5**). Fv/Fm (−18.80%), ΦPSII (−31.21%), ΦNPQ (−12.98%), NPQ (−47.88%), qP (−31.09%), and ETR (−30.28%) of the cut fronds sprayed with deionized water decreased relative to control, while ΦNO increased (+54.84%) (**Figure 6**). The relative losses of Fv/Fm (−12.91%), NPQ (−3.13%), and ETR (−10.06%) in the fronds treated with Put2.0 were lower than those with other treatments. The relative increases of ΦNO (+11.76%) in fronds treated with Put2.0 were lower than those upon other treatments.

**Figure 7:**
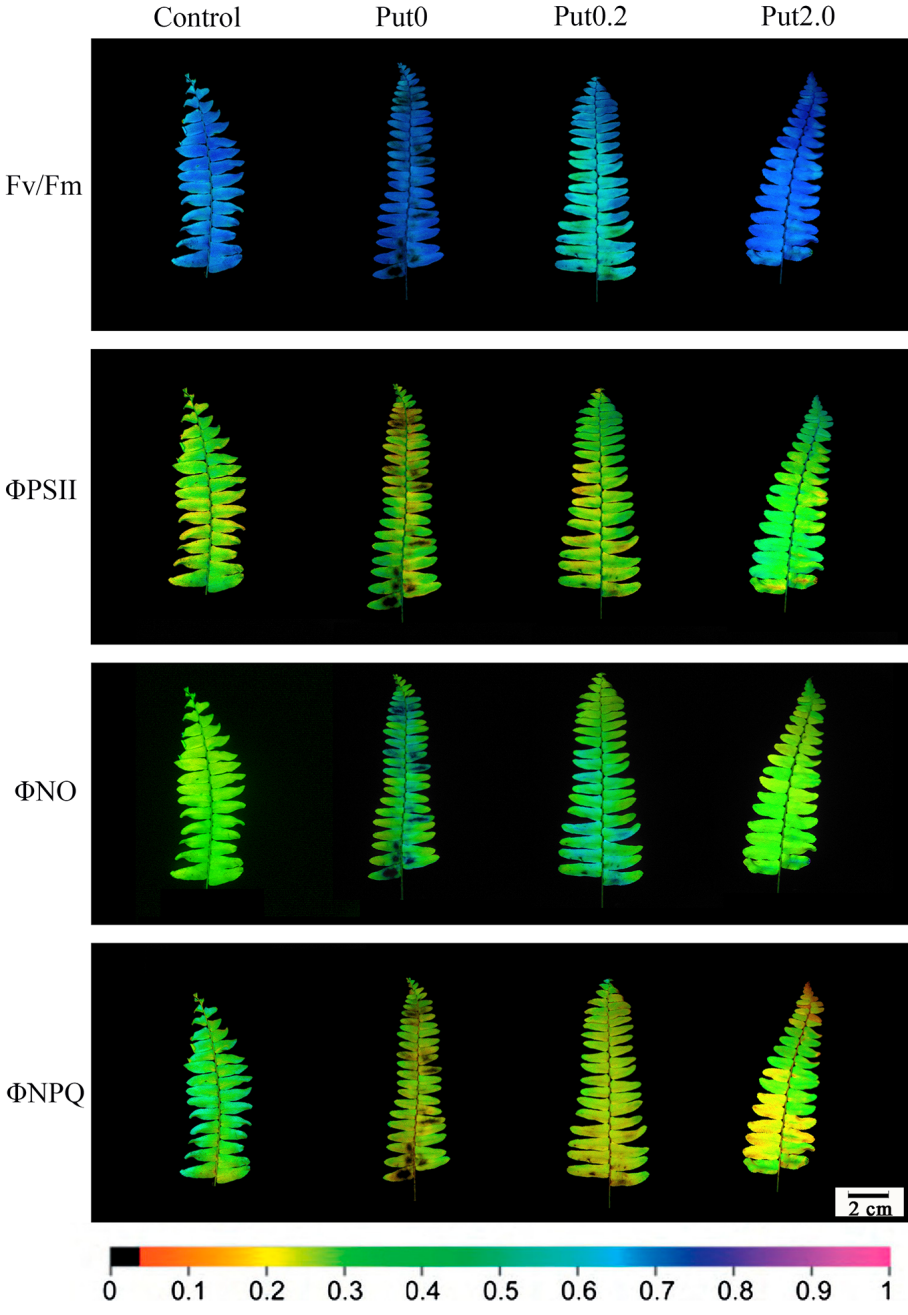
Detection of the effects of exogenous putrescine (Put) on cut foliage of *N. cordifolia* using a chlorophyll fluorescence imaging technique. Images of Fv/Fm, ΦPSII, ΦNO, and ΦNPQ, with actinic illumination of 196 µmol m^-2^ s^-1^ are shown. Each image in the same column represents the same frond. From left to right: Control, Put0, Put0.2, and Put2.0. The color scale at the bottom indicates values from 0 (black) to 1 (pink).

Chl fluorescence imaging presents an instantaneous overview of the fluorescence emission pattern of the whole leaf surface (Gorbe and Calatayud, 2012). The images of Fv/Fm, ΦPSII, ΦNO, and ΦNPQ showed that the fronds were stressed under Put0 after 10 days of deionized water spraying (**Figure 7**). Compared with Put0 treatment, foliar application of Put exerted significant mitigating effect. Distinct effects of Put0.2 and Put2.0 spraying on Fv/Fm, ΦNO, and ΦNPQ were found. Put2.0 seems to be more efficient in mitigating the negative effects of photoinhibition or photodamage than Put0.2. In the present study, heterogeneous distributions of these four parameters over the screened leaf area were observed especially in leaves sprayed with deionized water and Put0.2.

## Discussion

Half of the replicates in the cut foliage of sword fern treated with deionized water were beginning to shed pinnae after 10 days of treatment. Meanwhile leaf yellowing appeared, which is a normal symptom of leaf senescence. It has been reported that abscission of the leaflets of sword fern started from the fourth days in vase (Banthoengsuk et al., 2011). The discrepancy between this previous study and our result is probably due to the different ways for treatment. Spraying deionized water every day could postpone the water deficit and the following pinnae abscission and Chl degradation. In our experiment, Put (0.2 and 2.0 mM) alleviated Chl (Chl *a*, Chl *b*, and total Chl) loss significantly and higher concentration had a stronger effect (**Figure 2**). Our results are in agreement with some previous studies, in which application of Put on leaves of *Thymus vulgaris* (Mohammadi et al., 2018) and flower head of broccoli (Zheng et al., 2019) increased Chl *a*, Chl *b*, and total Chl content compared to those under natural senescence. It might be due to PAs could alleviate the loss of Chl in thylakoid membranes by stabilizing photosystem complexes during storage (Beigbeder et al., 1995).

During leaf senescence ROS level increases (Breusegem and Dat, 2006). In the present study, the O_2_^• −^ contents were similar between treatments, but the H_2_O_2_ was much higher under Put0 in comparison to control. Fronds sprayed with Put had lower increase of H_2_O_2_, suggesting it could play direct or indirect roles in scavenging H_2_O_2._ Both SOD and CAT are key antioxidant enzymes that have high ability of scavenging ROS under stress (Pan et al., 2006). SOD could convert O_2_^• −^ into H_2_O_2_ in cells and CAT converts H_2_O_2_ into H_2_O and O_2_ (Hossain et al., 2006; Liu et al., 2015). In the current study, SOD activities of the cut foliage under spraying treatments were not significantly different in comparison to control, probably due to the similar O_2_^• −^ contents between treatments. The activity of CAT was significantly elevated after 10 days in foliage sprayed with deionized water (Put0), suggesting the increase in CAT activity may be used to remove H_2_O_2_. It has been reported that an increase in PA content up-regulated the expression of genes encoding antioxidant enzymes and enhanced the activities of SOD, APX, and CAT under stress (Nayyar and Chander, 2004; Tang and Newton, 2005; Wi et al., 2006; Kuznetsov and Shevyakova, 2010). However, our results show that the SOD and CAT activities in Put-sprayed foliage were not significantly higher, even lower than those in Put0-sprayed foliage. Furthermore, the AsA content decreased with the leaf senescence, even lower in Put-sprayed foliage than that in Put0 treatment. These results indicate that Put detoxified the H_2_O_2_ directly or indirectly by triggering other pathways such as the γ-aminobutyric acid (GABA) shunt pathway (Groppa and Benavides, 2008; Palma et al., 2015; Palma et al., 2019).

Cellular membrane is very important for maintaining the osmotic balance of cells. ROS can attack the membrane and disrupt its integrity (Lim et al., 2007; Mohammadi et al., 2018). EL and MDA content are indicators of plasma membrane permeability and stability (Liu et al., 2015; Palma et al., 2016; Torabian et al., 2018). In the present study, foliage sprayed with deionized water had higher EL and MDA content while foliage sprayed with Put had slightly higher values or no significant difference compared to control, suggesting lower lipid peroxidation and higher membrane stability in Put-treated leaves (Wilhelmová et al., 2006). This result could be partly explained by the change of H_2_O_2_. In addition, this result could also be due to Put’s polycationic nature and it can directly bind to phospholipids to stabilize the membrane (Tiburcio et al., 1994; Bouchereau et al., 1999). During leaf senescence, the degradation of macromolecules and disassembly of cellular contents are often observed. In this study, Sp and Pro contents were decreased whereas Ss content was increased. This result indicates that protein degradation and Pro catabolism occurred after 10 days in vase (Zhang and Becker, 2015). However, Put spraying deterred this process. It is probably because Put is polycationic and could bind and stabilize these macromolecules (Tassoni et al., 1996). Another reason might be due to Put could enter the cell nuclear and produce thermospermine, then upregulate the prosurvival genes and activate ribosomal protein, finally produce more protein (Breeze et al., 2011; Moschou and Roubelakis-Angelakis, 2014). It has been proposed that the high level of Ss is the cause of senescence because of its repression of photosynthesis (van Doorn, 2008). Put spraying alleviated the increase of Ss content, suggesting that it could weaken the sugar signaling for anti-aging which is similar to the role of melatonin (Wang et al., 2013).

Generally, Fv/Fm, ΦPSII, and qP decrease under stresses and regulated-energy dissipation indicated by NPQ and ΦNPQ increase at first but decrease when the stress is more severe, while ΦNO increases (Gorbe and Calatayud, 2012). The decreased value of Fv/Fm often indicates photoinhibition (Thwe et al., 2014). In the present study, the fronds sprayed with deionized water (Put0) had a lower Fv/Fm compared with control, indicating the emergence of photoinhibition. The increase of ΦNO further confirmed that photodamage had occurred (Zhang et al., 2018). We found that the changes of Fv/Fm and ΦNO, indicators of photoinhibition and photodamage, respectively, were mitigated by the Put spraying. The values of ΦPSII, qP, and ETR were significantly reduced in fronds sprayed with deionized water (Put0) compared with control. The malfunction of the electron transport chain probably further induced the generation of H_2_O_2_ (Gupta et al., 2016). However, qP of the fronds sprayed with Put was not significantly different from qP in control. This result is in accord with a previous study showing that both ΦPSII and qP of cucumber plants under NaCl stress were reduced, while the reductions were alleviated to the control level by treatment with Put (Zhang et al., 2009). The mitigating effect of Put could be due to its polycationic nature, which induces the electrostatic screening, causes the stacking of thylakoid membrane and increases a spatial segregation of PSII and PSI (Barber, 1982; Ioannidis et al., 2006). In addition, Put can act as a permeant buffer to stimulate the synthesis of ATP, which can be used for PSII recovery after stress (Ioannidis et al., 2006; Ioannidis and Kotzabasis, 2007; Wu et al., 2019). The high value of NPQ indicates that plant has a high photo-protective ability and can protect itself from the damage *via* dissipation of excessive energy (Shu et al., 2013). In our study, we observed that NPQ in Put0 was significantly lower than that in control, suggesting the foliage was aging very serious after 10 days of treatment. Exogenous Put spraying significantly increased NPQ in the fronds compared with Put0. These results indicate that Put protects PSII against over-excitation by regulating heat dissipation probably through the aggregation of light harvest complex II (LHCII) (Pascal et al., 2005).

Chl fluorescence imaging is a non-destructive and easy operating technique that can rapidly detect spatial heterogeneities in fluorescence emission pattern and photosynthetic capacity across the leaf surface, even before visual injury appears (Omasa and Takayama, 2003; Calatayud et al., 2006; Gorbe and Calatayud, 2012). This technique is very useful for identifying early stress-induced damage. In the present study, the visible injury on frond surface was not severe and could not be detected easily by naked eye, except for fronds treated with Put0 (**Figure 1**), but the images of chlorophyll fluorescence parameters showed that the photochemical activity had already been inhibited and photodamage had occurred under both Put0 and Put0.2 treatments (**Figure 7**). In addition, there was obvious spatial heterogeneity in the fluorescence emission patterns over the frond surface under these two treatments. Mesophyll cells near the midrib in some leaflets under Put0 and Put0.2 treatments had lower Fv/Fm, ΦPSII, and ΦNPQ, but had higher ΦNO than mesophyll cells in other parts of the leaf. There is a report suggesting that leaf section next to the midrib has a more severe change of chlorophyll fluorescence parameters (Gorbe, 2009). They also find that the capacity of photoprotection of PSII, indicated by the ratio ΦNPQ/ΦNO decreases progressively as flower shoots wilt and vase life ends in cut rose flowers (Gorbe, 2009). Our results showed that the leaflets under Put0 and Put0.2 treatments lost the photoprotection ability due to the decreased ΦNPQ and increased ΦNO, while those sprayed with 2.0 mM Put still had this capability of dissipating excess energy. From the image of ΦNPQ, a significant difference among treatments was observed while the average values of this parameter among treatments were not different significantly. This result suggests that it is better to combine the qualitative and quantitative chlorophyll fluorescence analysis together to describe leaf senescence.

In conclusion, we investigated the effects of Put on senescence of cut foliage in sword fern. After fronds were detached from the plants, the Chl, Sp, and Pro contents were decreased, EL, MDA, and H_2_O_2_ contents were increased, plasma membrane was impaired, photochemical activity was inhibited, and photoinhibition and photodamage happened. Both 0.2 and 2.0 mM Put spraying extended the vase life of the cut foliage and alleviated the changes of parameters caused by senescence. In addition, 2.0 mM Put had a better mitigating ability than that of 0.2 mM. Leaf spraying of 2.0 mM Put for 10 days significantly ameliorated the losses of Chl, Sp, and Pro content (−10.72, −26.29, and −42.64%, respectively), followed by 0.2 mM Put (−27.36, −36.24, and −60.55%, respectively). Put spraying also improved photochemical capability and prevented membrane impairment as well as visible injury in comparison with cut fronds sprayed with deionized water. Leaf spraying of 2.0 mM Put greatly reduced the decline of the effective quantum yield of photochemical energy conversion in PSII (ΦPSII), the maximal quantum yield of PSII photochemistry measured in the dark-adapted state (Fv/Fm) and electron transport rate (ETR) (−7.89, −12.91, and −10.06%, respectively), and also inhibited the increases of EL, MDA, Ss, and H_2_O_2_ (+31.87, +6.43, +16.22, and +49.40%, respectively). Overall, Put played important roles in deterring the degradation of Chl, Ss, and Pro, detoxifying the H_2_O_2_, weakening the sugar signaling, mitigating the decline of photochemical activity, and postponing the leaf senescence. The present study gives new insights into effects of Put on leaf senescence and provides a strategy for preserving post-harvest cut foliage.

## Data Availability Statement

All datasets presented in this study are included in the article.

## Author Contributions

LZ and TW designed research. YQ, LJ, and SM conducted experiments. YQ, FX, and YC analyzed data. LZ, YQ, and TW wrote the manuscript. All authors contributed to the article and approved the submitted version.

## Funding

This research was supported financially by National Natural Science Foundation of China (No. 31711530648), the open funding of State Key Laboratory of Tree Genetics and Breeding (TGB2019004), Northeast Agricultural University of China (Academic backbone Project, No.18XG07), and Achimedes Foundation of Estonia.

## Conflict of Interest

The authors declare that the research was conducted in the absence of any commercial or financial relationships that could be construed as a potential conflict of interest.
